# Tudor staphylococcal nuclease drives chemoresistance of non-small cell lung carcinoma cells by regulating S100A11

**DOI:** 10.18632/oncotarget.3495

**Published:** 2015-03-26

**Authors:** Anna Zagryazhskaya, Olga Surova, Nadeem S. Akbar, Giulia Allavena, Katarina Gyuraszova, Irina B. Zborovskaya, Elena M. Tchevkina, Boris Zhivotovsky

**Affiliations:** ^1^ Institute of Environmental Medicine, Division of Toxicology, Stockholm, Sweden; ^2^ Ludwig Institute for Cancer Research Ltd, Karolinska Institutet, Stockholm, Sweden; ^3^ Department of Molecular and Developmental Medicine, University of Siena, Siena, Italy; ^4^ Institute of Biology and Ecology, Faculty of Science, Pavol Jozef Šafárik University in Košice, Košice, Slovakia; ^5^ NN Blokhin Russian Cancer Research Center, Moscow, Russia; ^6^ Faculty of Fundamental Medicine, ML Lomonosov State University, Moscow, Russia

**Keywords:** tudor staphylococcal nuclease, S100A11, phospholipase A_2_, non-small cell lung cancer, apoptosis

## Abstract

Lung cancer is the leading cause of cancer-related deaths worldwide. Non-small cell lung cancer (NSCLC), the major lung cancer subtype, is characterized by high resistance to chemotherapy. Here we demonstrate that Tudor staphylococcal nuclease (SND1 or TSN) is overexpressed in NSCLC cell lines and tissues, and is important for maintaining NSCLC chemoresistance. Downregulation of TSN by RNAi in NSCLC cells led to strong potentiation of cell death in response to cisplatin. Silencing of TSN was accompanied by a significant decrease in S100A11 expression at both mRNA and protein level. Downregulation of S100A11 by RNAi resulted in enhanced sensitivity of NSCLC cells to cisplatin, oxaliplatin and 5-fluouracil. AACOCF_3_, a phospholipase A_2_ (PLA_2_) inhibitor, strongly abrogated chemosensitization upon silencing of S100A11 suggesting that PLA_2_ inhibition by S100A11 governs the chemoresistance of NSCLC. Moreover, silencing of S100A11 stimulated mitochondrial superoxide production, which was decreased by AACOCF_3_, as well as *N*-acetyl-*L*-cysteine, which also mimicked the effect of PLA_2_ inhibitor on NSCLC chemosensitization upon S100A11 silencing. Thus, we present the novel TSN-S100A11-PLA_2_ axis regulating superoxide-dependent apoptosis, triggered by platinum-based chemotherapeutic agents in NSCLC that may be targeted by innovative cancer therapies.

## INTRODUCTION

Lung cancer (LC) is the leading cause of cancer-related deaths worldwide (http://globocan.iarc.fr). Lung cancer is classified as small cell lung carcinomas (SCLCs) and non-small cell lung carcinomas (NSCLCs). SCLC comprises about 15–20% of all cases, whereas NSCLC makes up to 80% of all bronchogenic cancers. NSCLC is divided into adeno-, squamous- and large cell lung carcinomas, accounting for about 40%, 30% and 10–15% of all lung cancer cases, respectively. Despite improvements in the efficacy of therapeutics, the overall five-year survival rate for lung cancer still hovers around 15% [1]. The initial response to therapy is also different among distinct forms of lung cancer. NSCLCs exhibit poor response even in the initial stage, and complete remission, concomitant with a higher degree of resistance to anti-cancer therapies, is rare. LC's resistance to available treatment modalities of radio- and chemotherapies have been considered as major clinical problems thus far. Accumulating data suggest that various treatments used to cure cancer usually inflict DNA damage and trigger apoptotic cell death response [2]. However, tumorigenic cells may increase DNA repair capacity as well as deregulate cell survival and apoptotic signaling circuits to impede proper therapy response. Since overall defects in apoptosis have been attributed as being prerequisite for cancer development [3], improved understanding of the targets and limiting factors that can affect tumor resistance are of higher importance for developing therapeutic efficacy to combat cancer.

A number of genes/proteins have been ascribed in positive regulation of apoptotic response upon chemotherapy. Likewise a plausible role of these genes/proteins in the low tumor response rate and poor clinical outcomes has been proposed but relatively few have been well characterized yet [4–7]. Staphylococcal nuclease and Tudor domain-containing protein 1 (SND1), also known as Tudor staphylococcal nuclease (Tudor-SN, TSN or p100), is a multifunctional evolutionary conserved protein that under physiological conditions regulates gene expression at both transcriptional and translational levels, and has been found to be associated with tumorigenesis [8, 9] This protein mainly acts as a transcriptional co-activator, chromatin and pre-mRNA processing/spliceosome regulator [10, 11]. Moreover, as an important component of RISC (RNA-induced silencing) complex, it is implicated in RNA interference and miRNA-mediated target regulation [12–14]. TSN has previously been shown to interact with many transcriptional factors such as STAT5, STAT6, c-Myb [15–17]. This association strongly emphasizes that unrestricted activation of TSN may result in hyperactivation of many such genes that eventually can lead to cancer initiation and progression.

Contemporary data has revealed TSN's role in carcinogenesis. It is listed among the genes highly upregulated in human colon carcinomas [8]. TSN has been identified as a conserved substrate of metacaspases harboring a consensus cleavage site for caspase-3-like enzymes that strongly supports its role in apoptotic cell death [18]. The role for TSN in promotion of breast cancer metastasis to lung was discovered recently [19]. TSN is highly expressed in prostate cancer tissues [20], where it intensifies even more along with the tumor's aggressiveness. However, TSN's role in lung cancer apoptotic response to chemotherapy is unclear and in-depth investigations remain to be carried out. Moreover, a mechanistic study is needed to highlight the potential regulators of lung cancer resistance that perturb their apoptotic capacity. Therefore, we aimed to investigate the role of TSN in the functioning of cell death machinery and its impact on the chemoresistance mechanisms of lung cancer.

Here we show for the first time that silencing of TSN in NSCLC cell lines results in strong induction of apoptosis upon treatment with cisplatin. Gene expression analysis performed using A549 NSCLC cells after silencing of TSN revealed several novel candidates for anti-cancer therapy downstream from TSN, such as S100A11, ATP6V1F, MDC1, BNIP3, etc., which are implemented in the cell death mechanism. Notably, none of these genes has been shown earlier to be linked to TSN signaling. Furthermore, here we show that S100A11 silencing sensitized NSCLC cells to treatment with cisplatin, oxaliplatin and 5-fluorouracil and suggest that S100A11 is a novel downstream effector of TSN that promotes resistance of NSCLC cells to chemotherapy. Overall, we demonstrate that TSN contributes to NSCLC chemoresistance by facilitating the expression of S100A11, which in turn inhibits phospholipase A_2_, leading to a decrease in mitochondrial superoxide production, thus suppressing mitochondrial superoxide-dependent apoptotic cell death. This suggests that TSN and S100A11 represent promising therapeutic targets to combat lung adenocarcinomas.

## RESULTS

### TSN is upregulated in NCSLC cells and tissues

**Figure 1:**
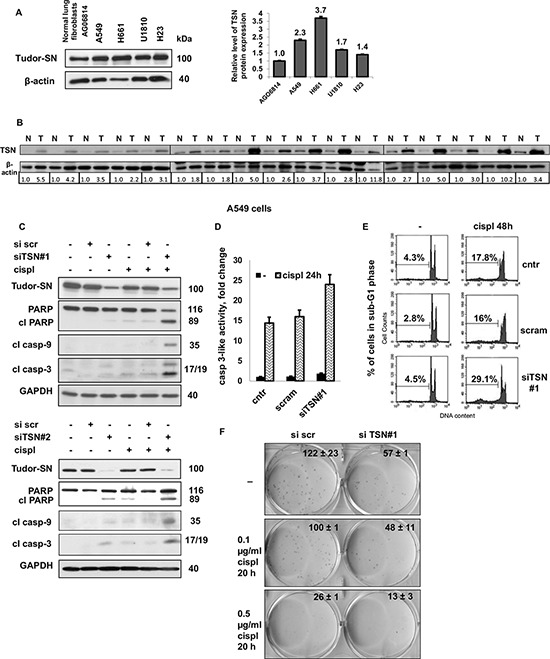
Tudor-SN is overexpressed in non-small cell lung cancer cells and patients’ tissues, and its silencing sensitizes NSCLC cells to cisplatin **A.** The level of TSN protein expression in normal human lung fibroblasts AG06814 and in NSCLC cell lines (A549, H661, U1810 and H23), *left panel*. Densitometric analysis of the western blotting bands for TSN normalized to *β*-actin is presented in the *right panel*. Results shown are the mean ± standard error of the mean of three independent experiments. **B.** The level of TSN protein expression in 17 pairs of NSCLC tumor **(T)** tissues and corresponding adjacent normal **(N)** tissues. Densitometric analysis of the western blotting bands for TSN normalized to *β*-actin is presented in the *lower panel*. **C.** Cleavage of PARP and processing of caspase-9 and -3 in A549 treated as indicated (cispl, 5 μg/ml for 24 hours). GAPDH was used as loading control. **D.** Caspase-3-like activity (fold change *versus* control) in A549 cells treated as indicated (cispl, 5 μg/ml for 24 hours). **E.** Representative DNA histograms displaying genomic DNA fragmentation in A549 cells treated as indicated (cispl, 5 μg/ml for 48 hours). The percentage of cells in sub-G1 fraction is indicated above the marked area in each diagram. **F.** Colony formation by A549 cells treated as indicated. The results are shown as the mean ± SEM of three independent experiments. *P* < 0.05. For details see “Materials and Methods” section. All data are representative of three independent experiments.

In order to investigate the expression of TSN in lung canCER cells, the level of protein expression was assessed in a panel of NSCLC cell lines (H661, A549, U1810 and H23), in normal lung fibroblasts AG06814 (Fig. 1A) and in clinical samples. Western blot (Fig. 1A, left panel) and densitometric analysis (Fig. 1A, right panel) revealed that the TSN protein was overexpressed (around a two-fold) in all cancerous cells compared to normal lung fibroblasts.

Moreover, strong upregulation of TSN protein, ranging from about a two- up to a 12-fold increase in NSCLC species compared to the adjacent normal tissues was detected in each of 17 pairs of lung adenocarcinomas and the corresponding normal tissues analyzed in this study (Fig. 1B). Densitometric analysis of TSN western blotting bands normalized to β-actin is shown in the lower panel of Fig. 1B for each pair of tumor (T) versus normal (N) tissue samples. These data indicate that a high level of TSN expression can potentially contribute to lung cell malignancy.

### Silencing of TSN by RNAi potentiates death of NSCLC cells upon treatment with cisplatin

In order to clarify whether high expression of TSN in LC cells might contribute to malfunction of apoptotic machinery and chemotherapeutic response of NSCLC cells, we manipulated the expression of TSN and exposed the cells to chemotherapeutic treatment. Platinum-based chemotherapeutic agents, such as cisplatin, are used in standard first-line therapy for LC, but the use of platinum compounds is limited by tumor resistance. Therefore, here we used cisplatin as a model chemotherapeutic drug to investigate the impact of TSN protein expression on NSCLC chemosensitivity. A549 cells were transfected with scrambled nontargeting siRNA (si scr) or two different TSN-specific siRNAs (siTSN#1; siTSN#2) and 48 hours post transfection the cells were treated with 5 μg/ml of cisplatin (cispl) for another 24 hours, then the cells were harvested and analyzed for protein expression, caspase-3-like activity and genomic DNA fragmentation (Fig. 1C–1E). The efficiency of TSN knockdown was evaluated by western blotting (Fig. 1C, Supplementary Fig. S1A, S1D). Treatment with cisplatin for 24 hours led to weak apoptotic response in nontransfected A549 cells and those transfected with nontargeting siRNA, as assessed by absence or low level of Poly (ADP-ribose)polymerase (PARP) cleavage, as well as processing of caspase-3 and -9 (Fig. 1C). The cells transfected with TSN siRNA demonstrated strong induction of apoptosis upon treatment with cisplatin, which was assessed by cleavage of PARP, as well as caspase-3 and -9 processing (Fig. 1C, TSN-specific siTSN#1, upper panel; siTSN#2, lower panel). TSN silencing also resulted in induction of caspase-3-like activity in A549 cells upon 24 hours treatment with cisplatin, which was increased 1.5-fold compared to TSN-expressing treated cells (Fig. 1D). Additionally, the quantitative analysis of apoptosis revealed a significant ~two-fold increase of the sub-G1 population in response to 48 hours treatment with cisplatin in A549 cells transfected with TSN siRNA compared to nontransfected cells or those transfected with scrambled siRNA (Fig. 1E). To examine long-term effect of TSN silencing on NSCLC cell survival clonogenic assay was performed (Fig. 1F) demonstrating significant decrease in A549 cells colony formation upon TSN downregulation by RNAi.

The effect of TSN silencing on apoptosis in response to cisplatin was not cell-specific, since in addition to A549 cells it was observed in two other NSCLC cell lines, H661 and U1810 (Supplementary Fig. S1). We found significant induction of apoptosis upon cisplatin treatment in TSN-knocked-down H661 cells compared to TSN-expressing cells, assessed by higher levels of processed caspase-9 and -3 and PARP cleavage (Fig. S1A), a four-fold increase in caspase-3-like activity (Supplementary Fig. S1B) and ~three-fold increased sub-G1 cell population (Supplementary Fig. S1C). The same or even more pronounced chemosensitizing effect of TSN knockdown was observed in U1810 cells. As shown in Supplementary Fig. S1, TSN silencing led to increased PARP cleavage in both cisplatin-treated and untreated U1810 cells (Fig. S1D) and resulted in a ~12-fold increase in caspase-3-like activity in response to 24 hours’ cisplatin treatment (Supplementary Fig. S1E). Overall, the data obtained indicate that high expression of TSN contributes to NSCLC chemoresistance and knockdown of TSN expression promotes a sustained sensitizing effect upon cisplatin treatment.

### Identification of potential molecular targets of TSN and their impact on cell chemoresistance and apoptosis

**Figure 2:**
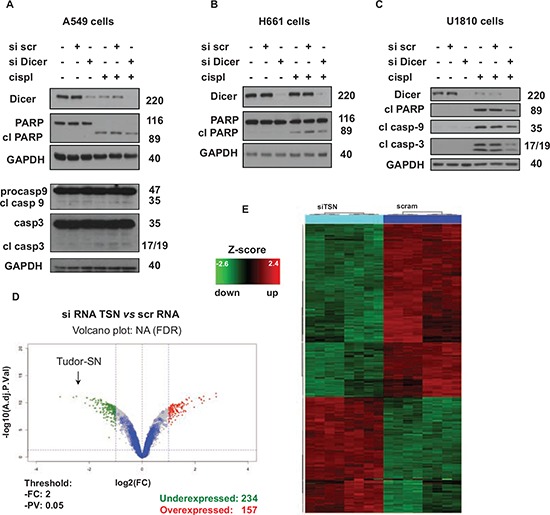
Effect of TSN silencing on chemosensitivity is related to its role in the regulation of gene expression **A.** Levels of Dicer, caspase-3, -9, and cleavage of PARP in A549 cells treated as indicated (cispl, 5 μg/ml for 24 hours). GAPDH was used as loading control. **B.** Cleavage of PARP in H661 cells treated as indicated (cispl, 5 μg/ml for 24 hours). GAPDH was used as loading control. **C.** Cleavage of PARP and processing of caspase-9 and -3 in U1810 cells treated as indicated (cispl, 5 μg/ml for 24 hours). GAPDH was used as loading control. The data are representative of three independent experiments. **D.** Volcano plot of microarray data. Blue spots represent low-intensity transcripts. **E.** Differentially expressed genes were used for hierarchical clustering of differentially expressed genes. For details see “Materials and Methods” and “Results” sections.

TSN is known to be one of the components of RISC (RNA-induced silencing complex) and is involved in RNA interference and miRNA-mediated target regulation [12–14]; therefore, we investigated whether the effect of TSN silencing on NSCLC sensitivity to cisplatin is related to TSN functioning as a part of the RISC complex. Using a specific siRNA pool, we carried out knockdown of endoribonuclease Dicer, another essential protein component of the RISC complex, and checked NSCLC cell sensitivity to cisplatin treatment. The effectiveness of Dicer silencing was evaluated by western blotting (Fig. 2A–2C). Knockdown of Dicer did not increase, but rather decreased the cell apoptotic response to cisplatin treatment, as assessed by the level of PARP cleavage in A549 and H661 cells (Fig. 2A, 2B), and also by the processing of caspase-3 and -9 in A549 and U1810 cells (Fig. 2A, 2C). Since Dicer silencing did not affect NSCLC sensitivity to cisplatin in a manner similar to TSN silencing in all three tested NSCLC cell lines (A549, H661 and U1810), we hypothesized that the role of TSN in NSCLC resistance to cisplatin was likely not related to its role in RNA interference machinery, but was rather mediated by transcriptional changes regulated by TSN. In order to identify potential molecular targets involved in NSCLC sensitization to cisplatin observed in this study, the global gene expression analysis was performed using an Agilent SurePrint G3 Human Gene Expression 8 × 60 K Microarray Kit. For this purpose, total RNA was extracted from TSN knocked-down as well as TSN-expressing A549 cells and subjected to microarray analysis. Widespread transcriptional changes were observed upon TSN knockdown: 391 unique genes demonstrated a greater than two-fold average change in expression, with 234 transcripts under- and 157 transcripts overexpressed compared to scrambled transfected control samples (Fig. 2D, 2E).

As TSN silencing affected the expression of a large number of genes, we next investigated which functional classes of genes were altered in the TSN knocked-down signature. Using the Ingenuity Pathways Analysis (IPA) program (Ingenuity Systems, Mountain View, CA, USA; http://www.ingenuity.com) and gene ontology category enrichment analyses, we selected several major networks containing genes that were closely associated with autophagy and apoptotic cell death as well as survival, DNA damage response and Ca^2+^ signaling (Table 1). The expression of shortlisted genes was further analyzed by q-RT-PCR, confirming microarray data (Supplementary Fig. S2); the primer sequences employed are shown in Supplementary Table S1. Indeed, the mRNA levels of the S100A11, ATP6V1F and MDC1 genes were strongly suppressed by TSN silencing (Supplementary Fig. S2A), while the mRNA levels of the BNIP, IGFBP2, ATG10, DRAM1, PDCD4, LAMP2 and BCL2L13 genes were significantly augmented compared to control samples (Supplementary Fig. S2B). Therefore, the number of TSN-regulated candidate genes involved in cell death machinery that were revealed in this study stress the strong implementation of TSN in the cell death mechanism and certify the role of TSN as an important mediator of chemoresistance.

**Table 1:** TSN's global regulation of genes involved in apoptotic and related signaling pathways

Gene bank accession ID	Gene Symbol	Major Functions
NM_005620	S100A11	Cell cycle progression and Differentiation
NM_014641	MDC1	DNA damage response, Apoptosis
NM_004052	BNIP3	Cell death
NM_000597	IGFBP2	Growth and Development
NM_031482	ATG10	Autophagy
NM_018370	DRAM1	Apoptosis
NM_145341	PDCD4	Apoptosis
NM_002294	LAMP2	Tumor cell Metastasis
NM_015367	BCL2L13	Apoptosis

### Silencing of TSN leads to downregulation of the S100A11 protein and silencing of S100A11 itself sensitizes NSCLC cells to cisplatin treatment

**Figure 3:**
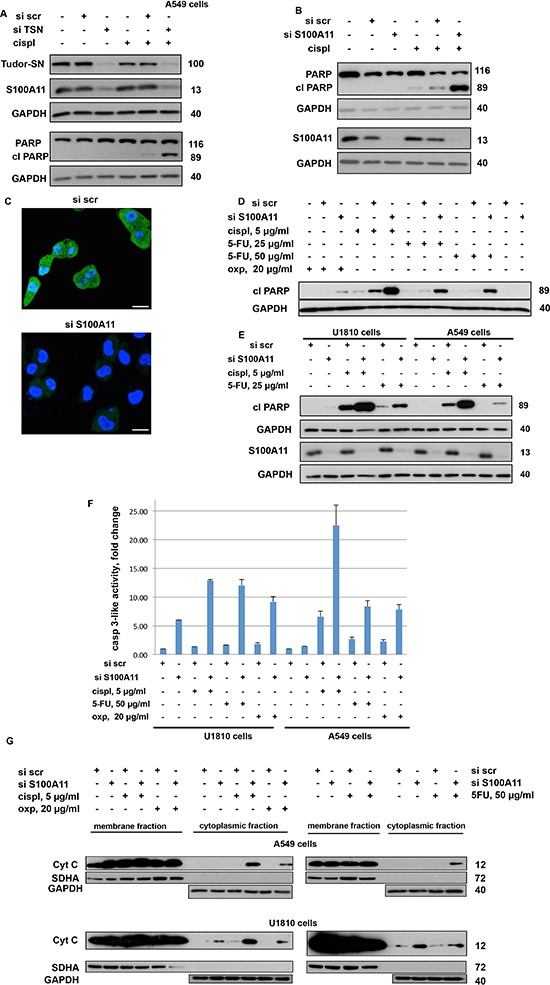
Silencing of TSN leads to downregulation of S100A11 and downregulation of S100A11 by RNAi chemosensitizes NSCLC cells similarly to the silencing of TSN **A.** Cleavage of PARP and S100A11 expression in A549 cells treated as indicated (cispl, 5 μg/ml for 24 hours). GAPDH was used as loading control. **B.** Cleavage of PARP in A549 cells treated as indicated (cispl, 5 μg/ml for 24 hours). GAPDH was used as loading control. **C.** Immunostaining of S100A11 in A549 cells transfected with nontargeting siRNA (si scr, *upper panel*) and S100A11-specific siRNA (si S100A11, *lower panel*) (72 hours post transfection). S100A11 is stained in green, while nuclei are stained in blue by Hoechst 33342 (Scale bar, 20 μm). **D.** Cleavage of PARP in A549 cells treated as indicated (24 hours with or without chemotherapeutic agent). GAPDH was used as loading control. **E.** Cleavage of PARP in U1810 and A549 cells treated as indicated (24 hours with or without chemotherapeutic agent). GAPDH was used as loading control. **F.** Caspase-3-like activity (fold change *versus* control) in U1810 and A549 cells treated as indicated (24 hours with or without chemotherapeutic agent). **G.** Cytochrome c (cyt c) level in membrane and cytoplasmic fraction of A549 (*upper panel*) and U1810 (*lower panel*) cells, treated as indicated (24 hours with or without chemotherapeutic agent). SDHA was used to verify the absence of mitochondria in the cytoplasmic fraction. GAPDH was used as loading control for cytoplasmic fraction. For details see “Materials and Methods” section. Different western blot images correspond to different exposure times of SuperRX X-ray films to the membranes treated with enhanced chemiluminescence reagents (to detect a noticeable signal from all protein bands under consideration within one membrane). Therefore, the band intensities are intended for comparison only within one membrane. All data are representative of three independent experiments.

Next we investigated which of the TSN-regulated genes contributes to NSCLC chemoresistance. The S100A11 gene demonstrates the most drastic decrease upon TSN silencing (Supplementary Fig. S2) and was previously reported to be overexpressed in NSCLC [21, 22]. Therefore, we examined S100A11 protein expression upon TSN silencing in the A549 NSCLC cells, and found significant downregulation of S100A11 at the protein level in both cisplatin-treated and non-treated cells (Fig. 3A).

Silencing of TSN significantly promoted apoptotic cell death in A549 cells treated with cisplatin for 24 hours, as assessed by the increased level of cleaved PARP (Fig. 3A). In order to test whether downregulation of the S100A11 protein is associated with the chemosensitizing effect of TSN silencing, A549 cells were transfected with scrambled nontargeting or S100A11-specific siRNA pools and 48 hours later exposed to cisplatin treatment. The efficiency of the S100A11 protein knockdown was confirmed by western blotting (Fig. 3B) and confocal microscopy of immunostained cells (Fig. 3C). We found that, similarly to the chemosensitizing effect of TSN silencing, S100A11 silencing also resulted in strong potentiation of apoptosis upon treatment with cisplatin, as evaluated by the increased level of cleaved PARP (Fig. 3B). These data indicate that a high level of S100A11 protein contributes to NSCLC resistance to cisplatin downstream from TSN. Therefore, we further focused our study on the role of S100A11 in NSCLC chemoresistance. The sensitizing effect of S100A11 silencing was not drug-specific since a similar increase in apoptosis was observed in response to other chemotherapeutic agents, such as oxaliplatin (oxp) and 5-fluorouracil (5-FU), as assessed by the increased level of PARP cleavage (Fig. 3D). Moreover, the sensitizing effect of S100A11 silencing was not specific to the A549 cells, since similar and even more pronounced chemosensitization was observed in another NSCLC cell line, U1810, as evaluated by the increased level of cleaved PARP (Fig. 3E), and an increase in caspase-3-like activity (Fig. 3F). S100A11 protein expression and the effectiveness of S100A11 silencing were similar in both cell lines (Fig. 3E).

Mitochondria are recognized as playing a key role in the regulation of the apoptotic process. The release of cytochrome *c* (cyt *c*) from mitochondria into the cytosol is considered to be decisive for the cell fate, as in the cytoplasm cytochrome *c* triggers apoptotic cascade [23]. We performed cell fractionation and examined the presence of cytochrome *c* in the cytoplasmic and membrane fractions by western blotting. The SDHA protein (succinate dehydrogenase complex, subunit A), expressed in mitochondria, was used as fractionation quality control, and GAPDH was used as a loading control for cytoplasmic fraction (Fig. 3G). S100A11 silencing resulted in an increased cytoplasmic level of cytochrome *c*, even in the absence of chemotherapeutic treatment in U1810 cells; 24 hours exposure of cells to cisplatin, oxaliplatin and 5-FU stimulated additional cytochrome *c* release, which was strongly potentiated in S100A11 knocked-down U1810 and A549 cells, compared to the corresponding S100A11 expressing cells (Fig. 3G). These findings suggest a novel role for S100A11 in promoting NSCLC chemoresistance.

**Figure 4:**
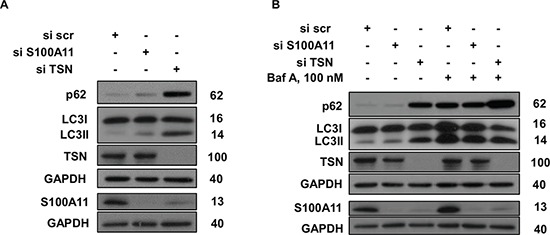
Silencing of TSN and S100A11 differently affects autophagy in NSCLC cells **A, B.** p62 accumulation and LC3 lipidation in A549 cells treated as indicated. GAPDH was used as loading control. For details see “Materials and Methods” section. The data are representative of three independent experiments.

Autophagy, a catabolic process regulating turnover of organelles and macromolecules, may promote cell death or preserve cell survival, depending on physiological context [24]. Interestingly, autophagy was not affected by silencing of S100A11 but was significantly suppressed by silencing of TSN, as assessed by LC3 lipidation and p62 accumulation in A549 cells (Fig. 4A). These data were further confirmed by assessment of autophagic flux using Bafilomycin A (Baf A) (Fig. 4B). Therefore, autophagy seems not to be involved in chemosensitization observed upon downregulation of S100A11, while widespread transcriptional changes observed upon TSN silencing may account for an additional role of TSN in autophagy regulation.

### Inhibition of phospholipase A_2_ abrogates the chemosensitizing effect of S100A11 silencing in a dose-dependent manner

**Figure 5:**
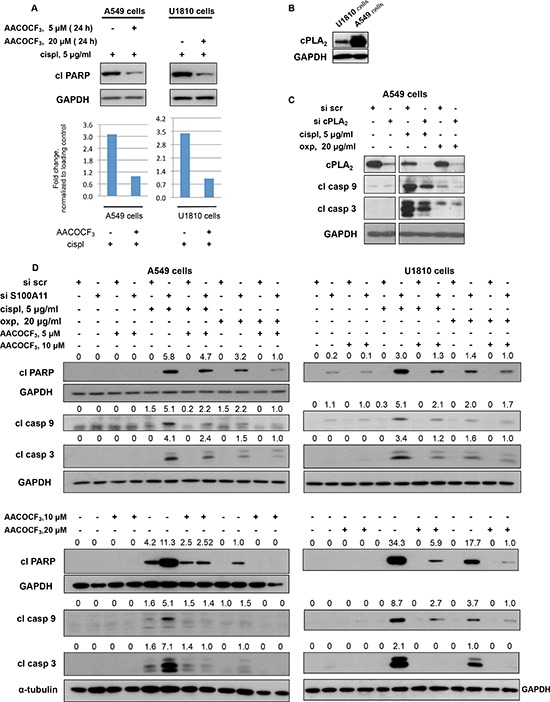
Chemosensitizing effect of S100A11 on NSCLC cell silencing involves PLA2 activity **A.** Cleavage of PARP in A549 and U1810 cells treated as indicated (*upper panel*). GAPDH was used as loading control. Densitometry data (normalized to GAPDH) (*lower panel*). **B.** cPLA_2_ expression in U1810 and A549 cells. GAPDH was used as loading control. **C.** cPLA_2_ expression and processing of caspase-3 and -9 in A549 cells treated as indicated. GAPDH was used as loading control. **D.** Cleavage of PARP and processing of caspase-9 and -3 in A549 cells treated as indicated (20 hours with or without chemotherapeutic agent). GAPDH and a-tubulin were used as loading control (panel showing corresponding GAPDH and a-tubulin bands are presented below the panel demonstrating the protein of interest). Densitometry values (normalized to GAPDH and a-tubulin) are presented above the corresponding western blotting panels. For details see “Materials and Methods” section. The data are representative of three independent experiments.

S100A11 has no intrinsic enzymatic activity, although it can bind to and modulate the activity of numerous cellular proteins [25]. S100A11 interactions were analyzed using Interactive pathway analysis of complex'omics data and S100A11-related pathways involved in apoptosis and cell resistance to cytotoxic treatment were selected for further analysis. In cell cytoplasm S100A11 was reported to interact with Annexin A1 and Annexin A2 [26, 27], known to inhibit phospholipases A_2_ (PLA_2_), a superfamily of enzymes involved in arachidonic acid (AA) release [28]. S100A11 was reported to facilitate Annexin A1-mediated inhibition of cytosolic phospholipase A_2_ (cPLA_2_) [27], which, among other PLA_2_ enzymes, in A549 cells was shown to mediate AA release and subsequent formation of its metabolites, which were inhibited by the PLA_2_ inhibitor, arachidonyl trifluoromethyl ketone (AACOCF_3_) [29]. Therefore, we investigated the effect of PLA_2_ inhibition by AACOCF_3_ on the chemosensitivity of NSCLC cells. In both A549 and U1810 cells, AACOCF_3_ significantly suppressed apoptosis, triggered by 24 hours cisplatin treatment (AACOCF_3_ was added to the cells 1 hour before cisplatin addition), as assessed by the decreased level of cleaved PARP (Fig. 5A, upper panel; densitometric analysis of the presented blots is shown in the lower panel of Fig. 5A). Thus, PLA_2_ activity, most likely, through increased liberation of AA and/or its metabolites contribute considerably to NSCLC chemosensitivity. cPLA_2_ may be involved in the AA release in both A549 and U1810 cells, since it is expressed in both cell lines, although at much higher level in A549 cells (Fig. 5B). Silencing of S100A11 may decrease the level of S100A11-Annexin complex and lead to less efficient inhibition of PLA_2_. Moreover, downregulation of cPLA_2_ by specific siRNA pool led to diminished apoptotic response of A549 cells to treatment with cisplatin and oxaliplatin (assessed by decreased levels of processed caspase-3 and –9), indicating that cPLA_2_ activity is involved in the regulation of NSCLC cells’ chemosensitivity (Fig. 5C).

Therefore, we tested whether inhibition of PLA_2_ activity by AACOCF_3_ would affect the chemosensitization of NSCLC cells upon S100A11 silencing. Consequently, A549 and U1810 cells were transfected with scrambled nontargeting or S100A11-specific siRNA pools in the presence of AACOCF_3_ at a concentration range previously reported to be sufficient to inhibit AA release in A549 cells [29], and 48 hours later were treated with cisplatin and oxaliplatin. As shown in Fig. 5D, AACOCF_3_ partially abrogated the chemosensitizing effect of S100A11 silencing upon treatment with both compounds, and the abolishment of this effect was much more pronounced with increasing concentrations of the PLA_2_ inhibitor. Therefore, S100A11 apparently exerts its promoting effect on NSCLC chemoresistance by facilitating the suppression of PLA_2_, since PLA_2_ inhibition rescued NSCLC cells from chemosensitization by S100A11 silencing in both cell lines.

### S100A11 silencing leads to elevated formation of superoxide radical, which is abrogated by the inhibition of PLA_2_

**Figure 6:**
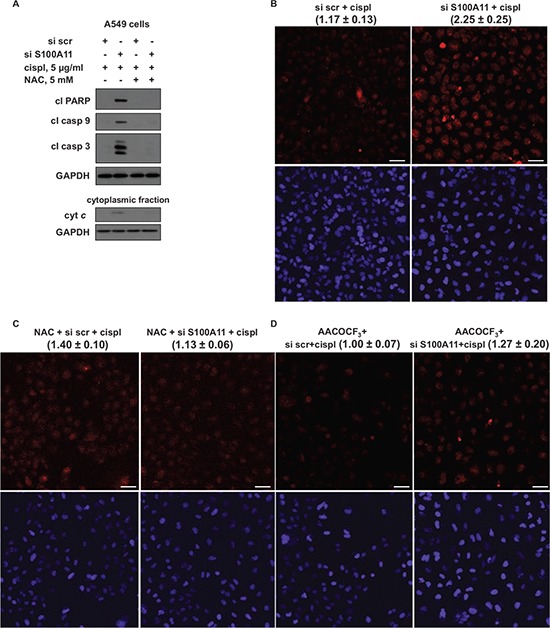
Chemosensitizing effect of S100A11 in NSCLC cells involves mitochondrial superoxide formation, which is abrogated by PLA2 inhibitor **A.** Cleavage of PARP and processing of caspase-9 and -3 in A549 cells treated as indicated (24 hours with or without chemotherapeutic agent) (*upper panel*). GAPDH was used as loading control. Cytochrome *c* (cyt *c*) release into the cytoplasm of A549 cells, treated as indicated (24 hours with or without chemotherapeutic agent) (cytoplasmic fraction, *lower panel*). GAPDH was used as loading control (panel showing corresponding GAPDH bands is presented below the panel demonstrating the protein of interest). The data are representative of three independent experiments. **B, C, D.** Representative images displaying staining of A549 cells, treated as indicated (12 hours with or without chemotherapeutic agent), with MitoSox Red mitochondrial superoxide indicator (*top picture*) and Hoechst 33342 (*bottom picture*) (Scale bar, 50 μm). The data were quantified using ImageJ software; the results are shown as the mean ± SEM of three independent experiments (the values are presented above each image). *P* < 0.05. For details see “Materials and Methods” section.

Arachidonic acid and its oxidized metabolites, as well as radicals generated as by-products during AA oxidation, may affect intracellular ROS production and subsequent signaling events regulating apoptosis [30–33]. To investigate whether S100A11 silencing chemosensitizes NSCLC cells by affecting intracellular ROS production, we exploited antioxidant *N*-acetyl-*L*-cysteine (NAC). A549 cells were transfected with scrambled nontargeting or S100A11-specific siRNA pools, 24 hours later the transfection mixture was replaced with complete growth medium containing 5 mM of NAC, then 24 hours later cisplatin was added for another 24 hours. As shown in Fig. 6A, NAC abolished the potentiation of apoptosis caused by S100A11 silencing. In addition, NAC abolished cytochrome *c* release stimulated by S100A11 silencing (Fig. 6A, lower panel). Confirming that the chemosensitizing effect of S100A11 silencing in NSCLC cells is mediated to a large extent by ROS production, we next examined the level of ROS formation in A549 cells. Mitochondria, being crucial regulators of apoptosis, also represent a major source of ROS in mammalian cells. Superoxide (superoxide anion radical, O_2_^•−^) is recognized as “primary” ROS; O_2_^•−^ is generated within mitochondria, further giving rise to other, “secondary” forms of ROS [34]. To assess ROS production we used MitoSox Red, a mitochondrial superoxide indicator, which is oxidized by superoxide in mitochondria. As shown in Fig. 6B, S100A11 silencing resulted in ~twofold increase in superoxide production upon treatment with cisplatin. Treatment with NAC did not induce a statistically significant difference in superoxide production in control cells (transfected with scrambled nontargeting siRNA pool), while it completely abrogated the rise in superoxide level in S100A11 knocked-down cells (Fig. 6C). In the presence of PLA_2_ inhibitor AACOCF_3_, the superoxide level was slightly decreased in control cells, while the rise in O_2_^•−^ production was diminished for ~80% (Fig. 6D). Our results indicate that in NSCLC cells treated with platinum-based chemotherapeutic agents, S100A11 silencing leads to higher PLA_2_ activity that drives enhanced mitochondrial superoxide production, which results in potentiation of apoptosis.

**Figure 7:**
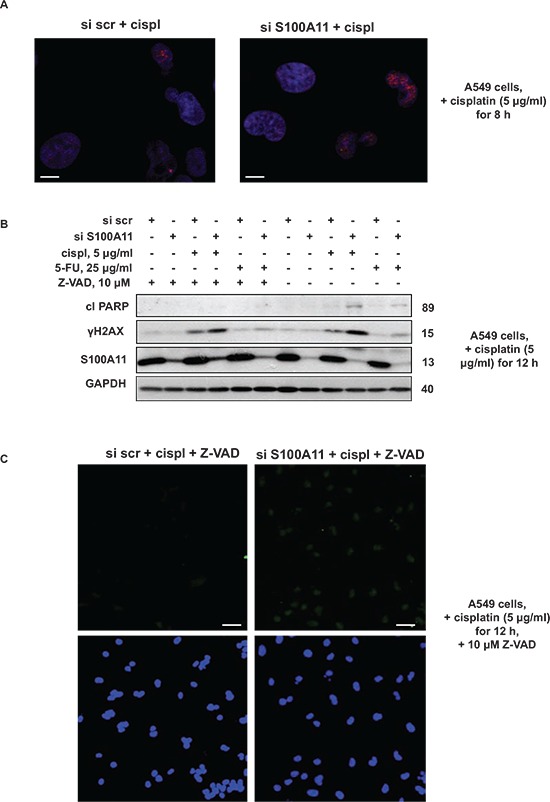
Silencing of S100A11 leads to increased formation of DNA strand breaks **A.** Immunostaining of γH2AX in A549 cells treated as indicated (Scale bar, 10 μm); **B.** Cleavage of PARP and γH2AX level in A549 cells treated as indicated. GAPDH was used as loading control. **C.** TUNEL staining of DNA strand breaks (*top image*) and Hoechst 33342 (*bottom image*) in A549 cells treated as indicated. (Scale bar, 50 μm). The data were quantified using ImageJ software; the results are shown as the mean ± SEM of three independent experiments (arbitrary units). *P* < 0.05. For details see “Materials and Methods” section. All data are representative of three independent experiments.

Superoxide, initially produced by mitochondria, is further dismutated to hydrogen peroxide, which, in turn, gives rise to highly reactive hydroxyl radicals [34], which can significantly affect cell signaling, causing damage to cellular proteins, lipids and DNA [35]. Here we observed enhanced formation of DNA double-strand breaks (DSB) in S100A11 knocked-down compared to S100A11-expressing NSCLC cells treated by chemotherapeutic agents. DNA DSB formation was assessed by the level of phosphorylated histone H2AX (γH2AX) using confocal microscopy (Fig. 7A) and western blotting (Fig. 7B). Immunostaining of paraformaldehyde-fixed A549 cells, exposed to cisplatin for 8 hours, revealed increased formation of γH2AX foci upon S100A11 silencing (Fig. 7A). In addition, the level of γH2AX was elevated in S100A11 knocked-down compared to S100A11-expressing cells treated with cisplatin or 5-FU for 12 hours (Fig. 7B). To ensure assessment of primary DNA damage (and not a result of DNA fragmentation due to induction of apoptosis), the pan-caspase inhibitor Z-VAD was added to the cells 1 hour before the chemotherapeutic agents. As shown in Fig. 7B, Z-VAD abolished the increased level of cleaved PARP in response to chemotherapeutic treatment, while the elevated level of γH2AX upon S100A11 silencing was preserved in the presence of this inhibitor. Formation of DNA strand breaks was further confirmed by TUNEL assay (Fig. 7C) in A549 cells treated with cisplatin for 12 hours upon inhibition of caspase activity by Z-VAD. These data indicate that chemosensitization of NSCLC cells upon S100A11 silencing (which leads to elevated mitochondrial superoxide formation) might, at least in part, result from enhanced DNA damage that potentiates DNA damage response signaling and apoptosis.

### S100A11 is overexpressed in NSCLC tissues

**Figure 8:**
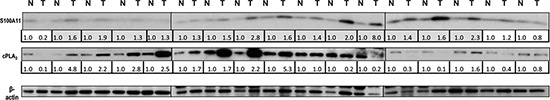
Expression of S100A11 and cytosolic phospholipase A2 (cPLA2) in NSCLC tumor tissues compared to corresponding normal tissues Expression levels of S100A11 and cPLA_2_ in 17 pairs of NSCLC tumors versus corresponding adjacent normal tissues. Densitometric analysis of the western blotting bands for the proteins of interest normalized to *β*-actin is presented in the *lower panel*. The rows corresponding to the samples where S100A11 was upregulated in tumor tissues compared to adjacent normal tissues are marked in grey.

Significant upregulation of S100A11 protein, ranging from about a 1.3- up to an 8-fold increase in NSCLC species compared to the corresponding normal tissues was detected in 14 out of 17 pairs (82%) of lung adenocarcinomas and the adjacent normal tissues analyzed (Fig. 8). Densitometric analysis of S100A11 and cPLA_2_ western blotting bands normalized to *β*-actin is shown in the lower panel of Fig. 8 for each pair of tumor (T) versus normal (N) tissue samples. In addition, overexpression of cPLA_2_ protein was observed in 64% of the NSCLC tumor species overexpressing S100A11 protein, compared to corresponding normal tissues (Fig. 8). It is important to note that in all clinical samples analyzed expression of TSN was upregulated (Fig. 1B). These data support the physiological significance of TSN-mediated regulation of S100A11, which affects PLA_2_ activity in NSCLC cells.

## DISCUSSION

Despite the recent progress in cancer therapy intrinsic and acquired tumor resistance remain a core issue for patients, clinicians and researchers. Therefore, it is of high importance to investigate mechanisms of chemoresistance in cancer cells, particularly in fatal and not-responsive to treatment tumors such as NSCLC.

As mentioned above, TSN (Tudor-SN), a multifunctional protein, is ubiquitously expressed and highly conserved from yeast to human suggesting its functional importance in varied cellular contexts; however, a comprehensive role of this protein in most of the tumors, including lung cancer, remains unknown. Here we found overexpression of the TSN protein in human NSCLC-resistant cell lines, as well as in NSCLC tissues (Fig. 1A, 1B). TSN was shown to represent a substrate for activated caspase-3 and to be important for the execution of apoptosis [18]; therefore, it was of interest to investigate the role of TSN in the apoptotic response of NSCLC cells to cytotoxic treatment. The strong sensitization of NSCLC cells to cisplatin upon TSN silencing observed in this study (Fig. 1C–1E, Fig. S1) emphasizes the importance of TSN in the regulation of apoptotic machinery and indicates the novel role for TSN as an essential mediator of NSCLC chemoresistance. NSCLC sensitization apparently resulted from transcriptional changes driven by TSN rather than possible deregulation of RNA interference within the RISC complex upon TSN silencing [12–14] (Fig. 2A–2C). Numerous gene expression alterations upon TSN silencing were revealed in this study (Fig. 2D, 2E, Table 1). None of the identified genes has been shown to be a possible TSN-regulated candidate previously, but many have been implicated in tumorigenesis, metastasis and cell death-related mechanisms [22, 36, 37]. In this work we demonstrate S100A11 as an important executor of NSCLC chemoresistance downstream from TSN (Fig. 3A, 3B).

S100A11 is highly conserved in vertebrates and expressed with high level in the lung and smooth muscle [38]. S100A11 has been shown to play diverse biological roles, being involved in the processes of endo/exocytosis, cell growth and inflammation, thus affecting the pathogenesis of various human diseases. Depending on cell type and conditions, S100A11 can be localized in the cytoplasm and/or nucleus and fulfills its biological function by interacting with other proteins and affecting their activity and/or cellular localization [25]. In various conditions it can interact with annexins A1 and A2, nucleolin, *β*-actin, Rad54B and p53 [38]. As shown in Fig. 3E–3F, the sensitizing effect of S100A11 silencing was similar in NSCLC A549 cells expressing wild type (wt) p53 and U1810 cells with p53 containing a frameshift mutation in the central p53 DNA-binding domain [39], referred to as not functional [40]. Therefore, we argue that the potential interaction of S100A11 with p53 is not a main driving mechanism promoting NSCLC chemoresistance.

Based on a tissue array containing 21 tumor types, the localization of S100A11 was shown to change from strictly nuclear in normal tissues to cytoplasmic and nuclear in many common cancers [41], which may contribute to enhanced proliferation of cancer cells. In our study, S100A11 demonstrated diffused staining in both the nucleus and cytoplasm of NSCLC A549 cells, the specificity of immunostaining was confirmed using S100A11-specific siRNA pool (Fig. 3C), and the distribution of S100A11 within the cell did not change noticeably upon cisplatin treatment. In cytoplasm, S100A11 can interact with calcium- and phospholipid-binding proteins Annexin A1 and Annexin A2, known to inhibit PLA_2_ enzymes, important regulators of cell signaling [27, 28, 42]. Here we present novel findings demonstrating the involvement of PLA_2_ in the S100A11-driven chemoresistance of NSCLC cells (both p53 wt and mutant), as the cPLA_2_ knock-down resulted in decreased apoptotic response to cisplatin and oxaliplatin, while PLA_2_ inhibitor abrogated chemosensitizing effect of S100A11 silencing upon treatment with these agents (Fig. 5). Thus, inhibition of cPLA_2_ by Annexin A1 was shown to be more effective [27] when it is complexed with S100A11, although S100A11 may potentially influence cPLA_2_ activity via other mechanisms. Thus, S100A11 interacts with Annexin A2, leading to plasma membrane repair [26]. Annexin A2 itself exhibits little or no cPLA_2_ inhibitory activity but it inhibits cPLA_2_ when complexed with S100A10; therefore, the complex Annexin A2-S100A11 may also possess cPLA_2_ inhibitory activity [43]. Moreover, S100A10, which shares structural similarities with Ca^2+^-bound S100A11, was shown to bind to and inhibit cPLA_2_ activity alone, independently of its association with Annexin A2 [44, 45]. Accordingly, S100A11 may itself exhibit cPLA_2_ inhibitory activity or influence S100A10 status within the cell, as S100 family proteins can form hetero oligomers [46].

PLA_2_ activity has been implicated in cell death modulation, with most reports indicating a cytotoxic role for AA [32] and many AA oxidized metabolites, such as 15-hydroxyeicosatetraenoic acid (15-lipoxygenase product) [47], some cytochrome P450 products [30] and 15-deoxy-Δ^12, 14^-prostaglandin J_2_ (15d-PGJ_2_), which was shown to act as a potent inducer of apoptosis in NSCLC A549 cells [33]. As a signaling molecule, AA was reported to affect multiple signaling pathways, such as JNK and p38MAPK [48, 49]; it also activates the sphingomyelinase-ceramide pathway, associated with cell death [50]. Furthermore, AA induces mitochondrial membrane permeability transition, release of cytochrome *c* and subsequent activation of apoptosis [51]. *In vitro* exposure of isolated mitochondria to AA led to increased generation of ROS by mitochondria, as a consequence of selective inhibition of respiratory chain complex I and III [52]. It was reported that AA-stimulated apoptosis was mediated by ROS production in rat hepatoma cells [53]. Moreover, oxidized AA metabolites, radicals, generated as by-products during AA oxidation, particularly via the mitochondrial P450 system, may stimulate mitochondrial ROS production, affect intracellular redox balance and induce apoptosis [30, 31, 33, 54]. We demonstrated the rise in mitochondrial superoxide radical production in cisplatin-treated NSCLC A549 cells upon S100A11 silencing, which was strongly abrogated together with the abolishment of chemosensitization, by NAC, as well as by the PLA_2_ inhibitor (Fig. 5, 6). Therefore, we suggest that S100A11 promotes NSCLC chemoresistance by facilitating PLA_2_ inhibition, consequently blocking AA release, which leads to the suppression of ROS-dependent apoptotic signaling.

Various processes can result from ROS-induced apoptotic signaling, such as altered cellular events, disruption of intracellular redox homeostasis, or irreversible oxidative modifications of lipids, proteins or DNA [35]. Thus, mitochondrial superoxide anion was shown to interact with voltage-dependent anion channels, leading to mitochondrial membrane permeabilization and cytochrome *c* release [55]. ROS-driven oxidation of mitochondrial phospholipid cardiolipin anchoring cytochrome *c* at the inner mitochondrial membrane facilitates its release into the cytoplasm and initiation of apoptosis [23, 56]. In accordance with these reports, in our study increased cytochrome *c* release upon S100A11 silencing was abrogated by NAC, together with the abolishment of elevated O_2_^−•^ production and chemosensitization, in NSCLC A549 cells treated with cisplatin (Fig. 6A). Furthermore, we observed enhanced DNA damage upon S100A11 silencing in A549 cells treated with chemotherapeutic agents (Fig. 7), which may result from elevated ROS production and, via DNA damage response signaling, contribute to the augmentation of apoptotic cell death.

S100A11 is overexpressed in several cancers and suggested to act either as tumor suppressor (in bladder and renal carcinoma) or oncogene (in prostate, breast and pancreatic cancer) [38]. In NSCLC, S100A11 is also overexpressed, and its high expression was associated with a higher tumor-node-metastasis stage and positive lymph node status [22]. Moreover, overexpression of S100A11 was demonstrated in NSCLC tissues and sera and the potential for S100A11 to be used as a biomarker for early NSCLC diagnosis was suggested [21]. These reports also support our observations showing regulation of S100A11 by TSN (Fig. S2A, Fig. 3A) which is overexpressed in NSCLC cell lines and in 82% of tumor samples (Fig. 1). The slight or no increase of S100A11 protein expression in three out of 17 samples analyzed in this study (18%) may be explained by a recognized heterogeneity of NSCLC tissues [57].

Thus, here we demonstrate the oncogenic function of S100A11, which is being a target of TSN involved in the regulation of chemoresistance of NSCLC cells. Altogether, this study revealed a novel mechanism driving the chemoresistance of NSCLC cells that involves overexpression of TSN promoting targeted upregulation of S100A11 expression, which leads to PLA_2_ inhibition and the suppression of ROS-dependent apoptotic signaling, particularly in response to platinum-based chemotherapeutic treatment. As cPLA_2_ was upregulated in 64% of NSCLC samples overexpressing S100A11, compared to normal tissues (Fig. 8), targeting TSN or S100A11 may be particularly beneficial for anti-cancer treatment in these cases. Inhibitors of S100 family proteins’ interactions start to emerge and may have important therapeutic implications [58]. NSCLC is a highly resistant to treatment and our findings suggest novel potential approach for the improvement of anti-cancer therapy for this tumor type.

## MATERIALS AND METHODS

### Human cell lines

Human NSCLC cell lines H23, A549, H661 (ATCC), U1810 (UU) [59] and normal human lung fibroblast cell line AG06814 (WI-38) [60] were used in this study. All NSCLC cells were cultured in RPMI medium, while AG06814 cells - in DMEM. Both media were supplemented with 10% (V/V) heat-inactivated fetal bovine serum (FBS), L-glutamine (2 mM), penicillin (100 U/mL) and streptomycin (100 μg/mL). Cell lines were maintained at 37^o^C, with 5% CO_2_ supply and 95% air-humidified conditions.

### Clinical material

Clinical samples were processed as described in [24].

### siRNA transfection/gene silencing

Cells were seeded at ~30% confluence prior to siRNA transfection. TSN-specific siRNAs were purchased from Invitrogen (Thermo Fisher Scientific, Carlsbad, CA, USA). All other siRNAs used in this study were purchased from Thermo Fisher Scientific Dharmacon^®^ (Dharmacon, Thermo Fisher Scientific Biosciences GmbH, St. Leon-Rot, Germany). siRNA transfection was performed according to the manufacturer's standard protocol (Dharmacon, Thermo Fisher Scientific, Lafayette, CO, USA).

### Treatment with inhibitors and chemotherapeutic agents

Cells were treated with the following chemotherapeutic drugs: 5 μg/ml of cisplatin (Hospira Nordic AB, Stockholm, Sweden), 20 μg/ml of oxaliplatin (Sigma-Aldrich, Stockholm, Sweden) and 25 or 50 μg/ml of 5-fluorouracil (Accord Healthcare Ltd, North Harrow, Middlesex, UK) for 15, 24 or 48 hours, as indicated in the text. Where indicated, cells were pretreated with AACOCF_3_ (Tocris Bioscience, Bristol, UK) or an equivalent amount of 95% ethanol for 48 hours before the chemotherapeutic agent was added to the medium. Z-VAD-fmk (Enzyme Systems Products, MP Biomedicals, Inc., Aurora, Ohio, USA) was added to cells where indicated 1 hour before the addition of the chemotherapeutic drug. Where indicated, cells were treated with Bafilomycin A for 5 hours.

### Flow cytometric analysis of sub-G1 population

The analysis of sub-G1 fraction of the cells was performed as described in [24]. The data obtained were evaluated using BD CellQuest Pro software by comparing the sub-G1 population in appropriate treated *vs* control samples.

### Gene expression analysis and target gene selection

To determine candidate genes, whose expression is altered upon TSN silencing, RNA was extracted from both mock-transfected and TSN siRNA-transfected A549 cells using a PureLink™ RNA Mini Kit (Life Technologies (Thermo Fisher Scientific), Carlsbad, CA, USA). Four biological replicates were considered to perform a gene expression analysis. Samples were hybridized to Agilent G4851A SurePrint G3 Human Gene Expression 8 × 60 K Microarray slides (listed with 27, 958 target gene RNAs and 7, 419 lincRNAs: Design ID 028004, Agilent Technologies, Santa Clara, CA, USA). All arrays were scanned by Agilent Microarray Scanner (G2565BA, Agilent Technologies, Santa Clara, CA, USA) and subsequently analyzed by Agilent Feature Extraction software 9.5.3.1 (Agilent Technologies) and Gene Spring GX12.0.2 software (Agilent Technologies, Santa Clara, CA, USA). Probes with an average of 2.0-fold changes were designated as TSN-regulated genes. The Ingenuity Pathways Analysis (IPA) program (Ingenuity Systems, Mountain View, CA, USA; http://www.ingenuity.com) was used to identify networks and canonical pathways of genes that appeared differentially expressed after silencing of TSN. With use of the Ingenuity knowledge database, the genes closely associated with cell death and survival pathways were selected for investigation.

### Real-time quantitative PCR analysis

Quantitative RT-PCR analysis was performed as in [24]. The primer sequences used in this study are shown in Supplementary Table S1.

### TUNEL assay

A549 cells were transfected as described above, plated on coverslips, treated with Z-VAD and cisplatin for 12 hours, and then fixed in 1% paraformaldehyde for 15 min. After washing with PBS, coverslips were incubated with TUNEL mix (Roche Diagnostics GmbH, Mannheim, Germany) for 1 hour at 37°C, washed with PBS, counterstained with Hoechst 33342, mounted on microscopy slides and analyzed using a Zeiss LSM 510 META confocal laser scanner microscope (Carl Zeiss MicroImaging, Göttingen, Germany).

### Clonogenic assay

A549 cells were transfected as described above, re-seeded into 6-well plate at 300 cells per well, treated with cisplatin at the indicated concentrations for 20 hours. After the treatment growth medium was replaced with the fresh medium. Ten days later cells were fixed (Acetic acid:Methanol, 1:7), stained with 0.5% Crystal Violet and washed extensively with PBS. Colony formation was quantified using a light microscope (Carl Zeiss MicroImaging, Germany).

### Western blotting

Western blotting was performed as described in [24]. Cell fractionation was performed as in [61]. The following primary antibodies were used: anti-Dicer, anti-cleaved PARP (detecting cleaved form of PARP), anti-cleaved PARP (detecting both full-length and cleaved PARP), anti-caspase-3, anti-caspase-9, anti-cleaved caspase-3, anti-cleaved caspase-9, anti-γH2AX, anti-cPLA_2_ (Cell Signaling Technology, Inc., Danvers, MA, USA); mouse monoclonal anti-TSN (FIT Biotech, Tampere, Finland); anti-LC3 (MBL International Corporation, USA); anti-*β*-actin, anti-*α*-tubulin (Sigma-Aldrich, Stockholm, Sweden); anti-G3PDH (Trevigen Inc., Gaithersburg, MD, USA); anti-cytochrome *c* (BD Biosciences, San Jose, CA); anti-p62 and rabbit polyclonal anti-S100A11 Abs (Santa Cruz Biotechnology, Inc., Santa Cruz, CA, USA). Protein molecular weights are presented on the right side of the corresponding western blotting panels. Densitometry of the protein signals was performed by using ImageJ software (http://rsbweb.nih.gov/ij/). The densitometry data were expressed as a fold change of the signal intensity compared to the lowest value, different from background readings, in the range of samples under comparison. The results shown are representative of three independent experiments.

### Confocal microscopy

Immunostaining of cells was performed as described in [24]. Primary antibodies (see Results section) were used at dilution 1:1000, secondary Alexa-Fluor-conjugated antibodies (Molecular Probes, Thermo Fisher Scientific, Carlsbad, CA, USA) were applied for 1 hour at room temperature (dilution 1:200). Nuclei were counterstained with Hoechst 33342 (10 μg/ml in PBS). Samples were examined using a Zeiss LSM 510 META confocal laser scanner microscope (Carl Zeiss MicroImaging, Göttingen, Germany).

### Superoxide-radical measurement

A549 cells were transfected, treated as described above and incubated with MitoSOX™ Red mitochondrial superoxide indicator (Molecular Probes, Thermo Fisher Scientific, Carlsbad, CA, USA) according to the manufactures instruction. After counterstaining with 10 μg/ml of Hoechst 33342 cells were immediately analyzed using a Zeiss LSM 510 META confocal laser scanner microscope (Carl Zeiss MicroImaging, Göttingen, Germany). The images obtained were quantified using ImageJ software (http://rsbweb.nih.gov/ij) and the data were calculated as corrected total cell fluorescence (CTCF), according to the equation CTCF = Integrated Density - (Area of selected cell × Mean fluorescence of background readings). The results were expressed as a fold change of the fluorescence intensity compared to the lowest value in the range of samples under comparison.

### Measurement of caspase-3-like activity

Caspase-3-like activity was assessed as described [61] and expressed as the fold change relative to corresponding controls. Results are presented as the mean ± standard error of the mean of three independent experiments.

### Statistical analysis

The results are presented as data from three independent experiments and expressed as the mean ± S.E.M (standard error of the mean). Statistical evaluation was performed using a nonpaired *t*-test.

## SUPPLEMENTARY FIGURES AND TABLE


