# Correction: Tudor staphylococcal nuclease drives chemoresistance of non-small cell lung carcinoma cells by regulating S100A11

**DOI:** 10.18632/oncotarget.8805

**Published:** 2016-04-18

**Authors:** Anna Zagryazhskaya, Olga Surova, Nadeem S. Akbar, Giulia Allavena, Katarina Gyuraszova, Irina B. Zborovskaya, Elena M. Tchevkina, Boris Zhivotovsky

Present: Due to a technical error during image processing, an incorrect high resolution version of Figure 7 was included with the manuscript.

Corrected: Correct Figure 7 is provided below. Authors sincerely apologize for this oversight.

Original article: Oncotarget. 2015 May 20; 6(14): 12156-73. doi: 10.18632/oncotarget.3495.

**Figure 7:**
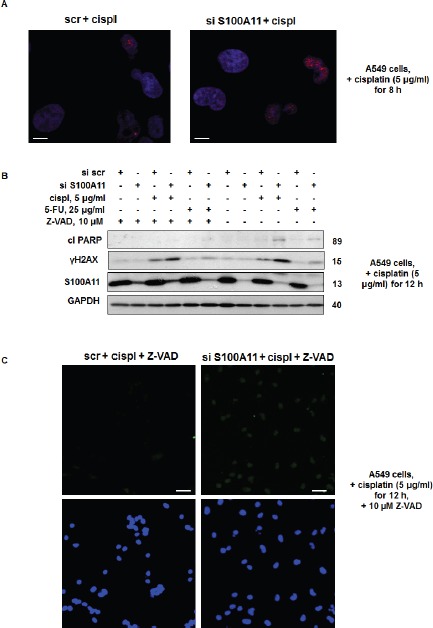
Silencing of S100A11 leads to increased formation of DNA strand breaks **A.** Immunostaining of γH2AX in A549 cells treated as indicated (Scale bar, 10 μm); **B.** Cleavage of PARP and γH2AX level in A549 cells treated as indicated. GAPDH was used as loading control. **C.** TUNEL staining of DNA strand breaks (*top image*) and Hoechst 33342 (*bottom image*) in A549 cells treated as indicated. (Scale bar, 50 μm). The data were quantified using ImageJ software; the results are shown as the mean ± SEM of three independent experiments (arbitrary units). *P* < 0.05. For details see “Materials and Methods” section. All data are representative of three independent experiments

