# Supplementary material for: Tudor staphylococcal nuclease drives chemoresistance of non-small cell lung carcinoma cells by regulating S100A11
Source: Oncotarget. 2015 Mar 26;6(14):12156–73. doi: 10.18632/oncotarget.3495 (PMC4494929; doi:10.18632/oncotarget.3495)
Supplement: Supplementary file 1 [file oncotarget-06-12156-s001.pdf]

SUPPLEMENTARY FIGURES AND TABLE

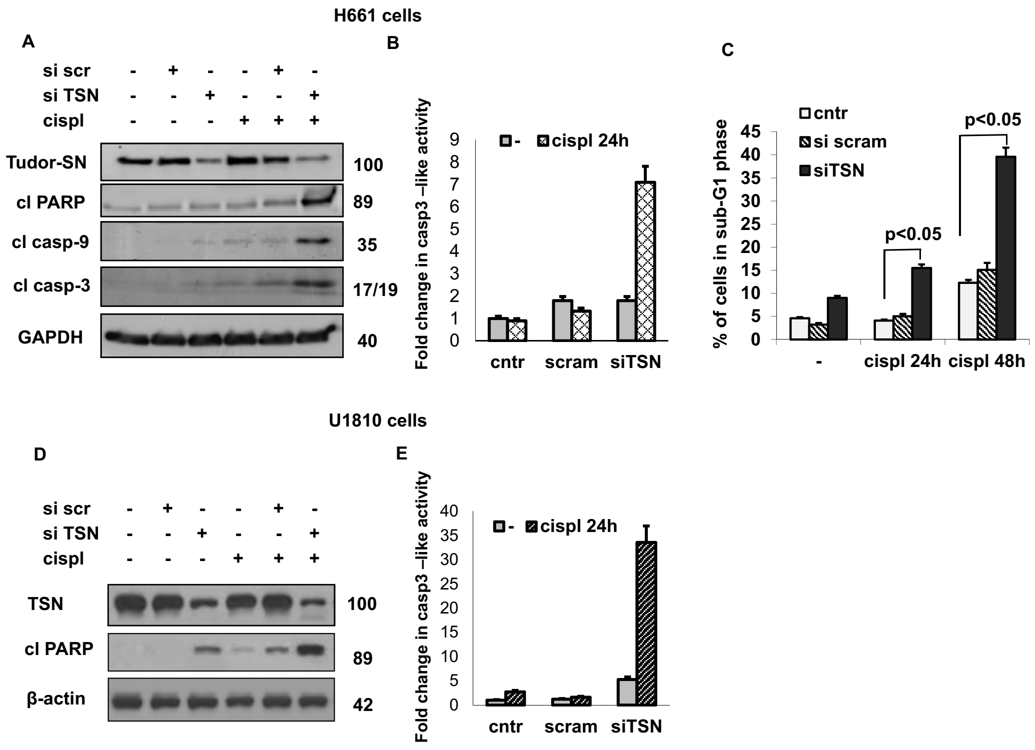

Supplementary Figure S1: Silencing of TSN sensitizes cells to cisplatin treatment in H661 and U1810 NSCLC cell lines. A. Cleavage of PARP and processing of caspase-9 and -3 in H661 cells treated as indicated (cispl, 5 μg/ml for 24 hours). GAPDH was used as loading control. **B.** Caspase-3-like activity (fold change *versus* control) in H661 cells treated as indicated. **C.** The percentage of H661cells in the sub-G1 fraction after indicated treatment. Results shown are the mean ± standard error of the mean of three independent experiments. $P < 0.05$. **D.** Cleavage of PARP in U1810 cells treated as indicated (cispl, 5 μg/ml for 24 hours). β-actin was used as loading control. **E.** Caspase-3-like activity (fold change *versus* control) in U1810 cells treated as indicated. For details see "Materials and Methods" section. All data are representative of three independent experiments.

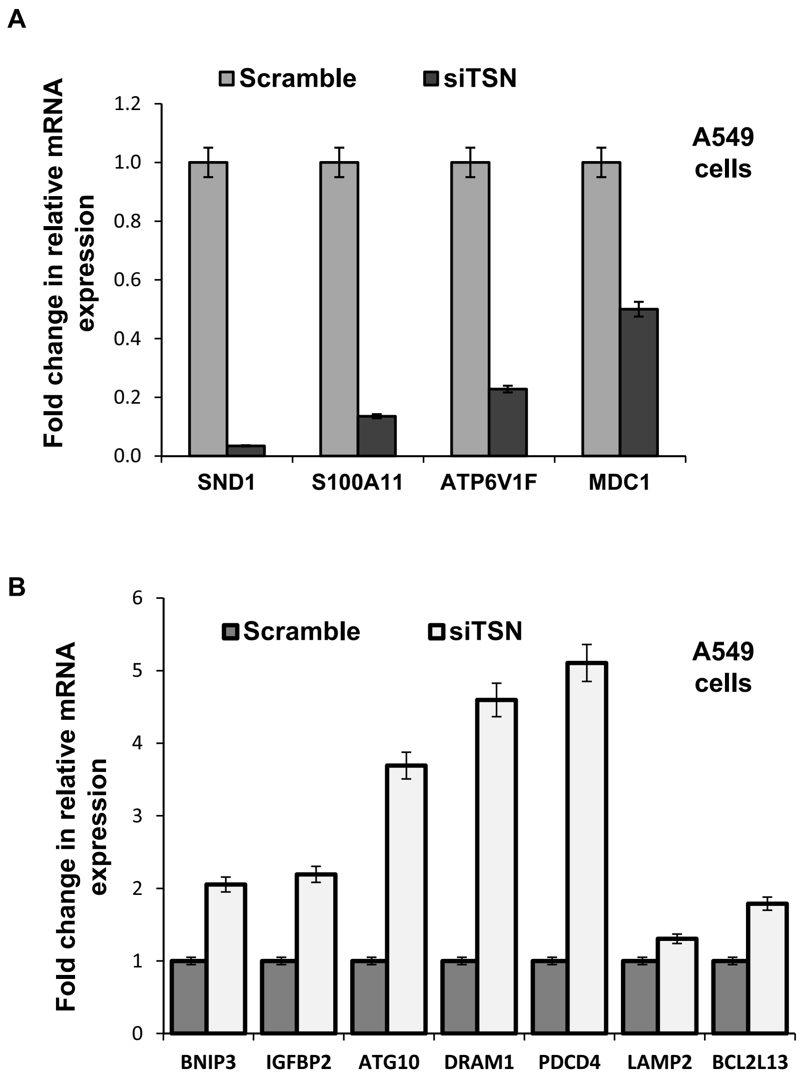

Supplementary Figure S2: Validation of microarray data by q-RT-PCR. A. mRNA level of genes (fold change *versus* control), underexpressed upon TSN silencing in A549 cells. **B.** mRNA level of genes (fold change *versus* control), overexpressed upon TSN silencing in A549 cells. Results shown are the mean ± standard error of the mean of three independent experiments. For details see "Materials and Methods" and "Results" sections.

Supplementary Table S1. Primer sequences employed in q-RT-PCR analysis to ascertain candidate gene's up- or downregulation upon silencing of TSN

Gene Symbol	Primers Sequences (5′ to 3′)
**S100A11**	Forward: GCATCGAGTCCCTGATTGCT<br>Reverse: GGGTCCTTCTGGTTCTTTGTGA
**ATP6V1F**	Forward: AGTGATCGGAGACGAGGACAC<br>Reverse: CCGGTTTAGAAATTGCCGG
**MDC1**	Forward: GAAGGCTGCTAGAGGGCTATGAG<br>Reverse: GAGGCTTATAGGACCGAGGCA
**BNIP3**	Forward: GGCTCCTGGGTAGAACTGCA<br>Reverse: AGCCCTGTTGGTATCTTGTGGT
**IGFBP2**	Forward: CAGGTTGCAGACAATGGCG<br>Reverse: CACGGCCAGCTCCTTCATAC
**ATG10**	Forward: CCGAGCGGAGAGGGTTATCA<br>Reverse: CCTTTGATGGTCTCCATTCCCA
**DRAM1**	Forward: GATGGTCATCTCTGCCGTTTCT<br>Reverse: AAGGCCACTGTCCATTCACC
**PDCD4**	Forward: CCCGAGGGATTCTGAAGGAAG<br>Reverse: AAGCACCATGGAGAATTAGGGAA
**LAMP2**	Forward: CAGACTGTTTCAGTGTCTGGAGC<br>Reverse: TGCACTGCAGTCTTGAGCTGT
**BCL2L13**	Forward: TGACCGTCACACTTCTCCAGTG<br>Reverse: CCCGAAATGCCTGATATGTCAC
**ACTIN BETA**	Forward: GCTGTGCTATCCCTGTACGC<br>Reverse: GAGGGCATACCCCTCGTAGA
